# The Penn Medical University; an Open Letter from “The Historian”

**Published:** 1881-08

**Authors:** 


### THE PENN MEDICAL UNIVERSITY.

Editor of "The Homœopathic Physician."

*Dear Sir:* My attention has been called to an editorial in the May number of your journal, under the following heading: "Perverted; The History of Homœopathy," in which you criticise the history of the Penn Medical University, as given in the historical volume of the World's Convention. The last sentence of this editorial reads: "If we are shown to be in error, we will most willingly acknowledge it."

Inasmuch as this editorial contains numerous errors—errors which can only be accounted for on the theory that your information came from a very unreliable source, I have deemed the matter of sufficient importance to make the following corrections:

1st. It can be hardly just to say that there was an attempt to "palm off the Penn Medical University as a Homœopathic Medical School," when it was clearly stated in the history, that allopathy, as well as homœopathy, was taught in the institution.

2d. You state "that the Penn Medical University was a re-organization of the Pennsylvania University of Medicine." I reply (1) there never was such an institution as the latter, and (2) the Penn Medical University was a re-organization of no school, and its charter, as obtained in 1853, was absolutely new.

3d. It is stated that this institution "never pretended to teach homœopathy."

On the contrary, the announcements not only state that "lectures on homœopathy would be delivered," and text-books on the same recommended, but, in fact, courses were actually given by the Professors of Pathology, Botany and Materia Medica.

4th. You say the history gives an "ideal faculty;" that "such a faculty never existed;" that "it is a piece of patch-work." The faculty as given, was copied verbatim from the announcement of 1854–55, and every man on the list had been regularly appointed, and every one filled his position, with one exception only.

5th. You say "that the claimed teachers of homœopathy in the Penn Medical University, could not have done so, is proven by their teaching afterwards in an avowedly eclectic college." In reply, none of the teachers of *homœopathy* in the Penn Medical University, ever afterwards taught in an eclectic college.

6th. The statement made in the history, "that a large proportion of the graduates of this institution are now practicing homœopathy in different parts of the country," if true, is certainly some evidence that they were probably taught it in their Alma Mater. The historian might have added, that of the old-school physicians who accepted positions in the faculty with prejudices against homœopathy. Six became converted while in the institution, and are now practicing that system. Finally, that the Penn Medical University claimed to teach homœopathy, was well known to the profession at the time; homœopathic physicians sent their students there with that understanding, and the "regulars" denounced it for the same reason; and one member of the faculty was expelled from the Philadelphia County Medical Society, for associating himself with homœopathic physicians. Whether it were wise to attempt thus to teach both systems of practice in the same institution, is a question about which there might be a difference of opinion, but the experiment was made and forms a part of the history of homœopathy, in Philadelphia. Up to the time of its closure, in 1864, the history of this institution was without reproach. With its later associations with disreputable institutions and the illegal attempt to revive the same, some ten years later, wholly under allopathic management, the history of homœopathy has nothing to do, neither has

Yours,

THE HISTORIAN.

[We publish the "corrections" of "The Historian" gladly, so that our readers may judge, after having read all that can be said in favor of the Penn Medical University, whether or not it were a true homœopathic institution. Its claim to such an honor rests on lectures given by the two professors of botany and pathology. Presumably, the rest of the faculty lectured on allopathy entirely; and these two, we presume, did the same at certain hours or days. We ask, what kind of homœopathy would a man teach who lectured one day on allopathy, the next day on homœopathy? This course is followed in nearly all the avowedly eclectic schools in the land. Why not include them in the history of homœopathy? The allopaths repudiate all connection with so-called eclectic institutions, as well as with the homœopathic, therefore, their disapproval proves nothing. The man who conducted the "Penn Medical University" in its latter days, was a member of the faculty when it flourished as a *homœopathic college!*

We ask, has homœopathy sunk so low that it claims as its own such an institution as this Penn Medical University?

We have said enough to show that this institution cannot be rightfully foisted upon homœopathy. We made our protest against this "University," because we deemed it our duty to warn the institute and the profession against it. The institute has swallowed too many camels to strain at such a small gnat as this institution. We expected this.

Of one thing we are sure; this institution would *never* have been placed in the "historical volume" had Carroll Dunham lived.—EDITOR.]

